# Benzodiazepines Reduce Relapse and Recurrence Rates in Patients with Psychotic Depression

**DOI:** 10.3390/jcm9061938

**Published:** 2020-06-21

**Authors:** Hiroki Shiwaku, Masako Fujita, Hidehiko Takahashi

**Affiliations:** Department of Psychiatry and Behavioral Sciences, Tokyo Medical and Dental University Graduate School, 1-5-45, Yushima, Bunkyo-ku, Tokyo 113-8519, Japan; 130732ms@tmd.ac.jp (M.F.); hidepsyc@tmd.ac.jp (H.T.)

**Keywords:** major depressive disorder, benzodiazepines, relapse, recurrence

## Abstract

The long-term use of benzodiazepines is not recommended for the treatment of major depressive disorder (MDD) due to the risk of adverse effects, including dependence, falls, dementia, mortality and the lack of evidence of effectiveness for symptoms other than anxiety. However, there are many patients with MDD for whom antidepressants are co-administrated with benzodiazepines. This study aimed to identify whether the use of benzodiazepines is associated with a lower risk of relapse or recurrence of MDD in some patients, and the characteristics of these patients. Kaplan–Meier survival analysis was used to quantify the relapse and recurrence of MDD in 108 patients with MDD who achieved remission during hospitalization. Among them, 26 patients had been diagnosed with severe MDD with psychotic features. There was no significant difference in the rate of relapse/recurrence between patients with and without benzodiazepines when all patients were analyzed together. However, among the 26 patients with psychotic depression, 21.2% in the benzodiazepine group and 75.0% in the non-benzodiazepine group experienced relapse (log rank *p* = 0.0040). Kaplan–Meier survival analysis revealed that this effect was dose-dependent. The adjunctive use of benzodiazepines may reduce relapse/recurrence rates in patients with severe MDD with psychotic features.

## 1. Introduction

Preventing the relapse or recurrence of major depressive disorder (MDD) is a major clinical goal. The standard approach is to continue medication with antidepressants for a sufficient period after the achievement of remission [1,2,3]. However, even with optimal medication adherence, there are some patients who are prone to relapse [1,2,3]. It is necessary to develop appropriate treatment strategies for such patients.

Benzodiazepines are generally not recommended for the treatment of depression due to the high risk of dependency [4]. Benzodiazepines are also associated with other adverse effects, such as increased risk of falls [5], increased incidence of dementia [6,7] and increased mortality [8]. Although the use of benzodiazepines with antidepressants results in fewer treatment dropouts and greater improvement in depressive symptoms in the first 4 weeks of treatment than the use of antidepressants alone, there is no evidence that it is better over the long term [9,10]. Similarly, although clinicians occasionally use benzodiazepines alongside antidepressants to alleviate anxiety or persistent sleep disorders, there are no systematic data demonstrating the effectiveness of benzodiazepines in the maintenance of remission in depression [11]. However, a substantial number of patients with depression use benzodiazepines over a long time frame [12,13]. Thus, it is important to determine whether the use of benzodiazepines is associated with a lower risk of relapse or recurrence of MDD in some patients, and to identify the characteristics of these patients, because it will minimize the use of benzodiazepines and aid the development of appropriate treatments for patients who are prone to relapse. 

To address these discrepancies between the literature and clinical practice, we used data of the clinical course after discharge of patients hospitalized with MDD at the Tokyo Medical and Dental University Hospital, and described a subgroup of patients in which the use of benzodiazepines is associated with the rate of relapse and recurrence.

## 2. Materials and Methods

### 2.1. Ethics Statement

The study was approved by the Institutional Ethics Committee of Tokyo Medical and Dental University (M2017-261), and was in accordance with the Helsinki Declaration and Ethical Guidelines for Medical and Health Research Involving Human Subjects in Japan. All the participants signed their written informed consent.

### 2.2. Study Cohort

All patients who were hospitalized between 1 January 2012 and 31 December 2017 at the department of psychiatry at Tokyo Medical and Dental University Hospital were screened. The inclusion criteria were a diagnosis of MDD or recurrent MDD at hospital admission and full remission at discharge, according to the Diagnostic and Statistical Manual of Mental Disorders-IV-TR (DSM-IV-TR). Remission was defined as a Hamilton Depression Rating Scale (HAMD-17) score of <8 for two consecutive weeks. Exclusion criteria were comorbid diagnoses of organic brain damage, personality disorder, developmental disorder, intellectual disability, or somatic symptom disorder. These comorbidities were excluded because they influence relapse rate. Patients diagnosed with bipolar disorder after discharge from hospital, and patients who relapsed due to reducing antidepressant medication or other psychotropic agents, were also excluded. All patients, except two who relapsed due to the cessation of antidepressants by themselves, were given the same medication throughout the year. There were no cases in which benzodiazepines were reduced or newly prescribed. This excludes the possibility of withdrawal symptoms influencing the relapse/recurrence data.

### 2.3. Outcomes

The primary outcome measure was the time to relapse/recurrence of depression. Patients were followed up every month for 12 months after hospital discharge. Relapse/recurrence was defined as an episode meeting the DSM-IV criteria for a major depressive episode and a HAMD-17 score of ≥14, and determined using retrospective data collected from patient charts completed every month.

### 2.4. Benzodiazepine Score

Benzodiazepine score (BZD score) was calculated using the following formula:

(1) $$BZD\;score=\overset{n}{\underset{i=1}{\sum }}\frac{D_{i}H_{i}}{S_{i}}$$

where *D* is drug dose (mg), *H* is half-life (hours) and *S* is drug strength. Drug strength was obtained from the table of dose equivalence of benzodiazepines in a previous report [14]. For example, 1.5 mg of lorazepam corresponds to a BZD score of 15 (*D* = 1.5 mg, *H* = 12 h, *S* = 1.2). *D*/*S* was multiplied by the half-life, since *S* (drug strength) reflects the short-term clinical anxiolytic effect and does not take into account long-term cumulative pharmacokinetic effects.

### 2.5. Statistical Analysis

Statistical analysis was performed using Prism 8 (GraphPad) software and EZR (Saitama Medical Center, Jichi Medical University, Saitama, Japan). Kaplan–Meier curves and log rank tests were used to examine the difference in depression relapse and recurrence between MDD patients who received benzodiazepines and patients who did not. Among patients with severe MDD with psychotic features, BZD scores and patient characteristics were compared between those with and without relapse/recurrence using a Mann–Whitney U test and Fisher’s exact test. Cox regression analysis was performed using EZR.

## 3. Results

### 3.1. Participants Characteristics

**Figure 1:**
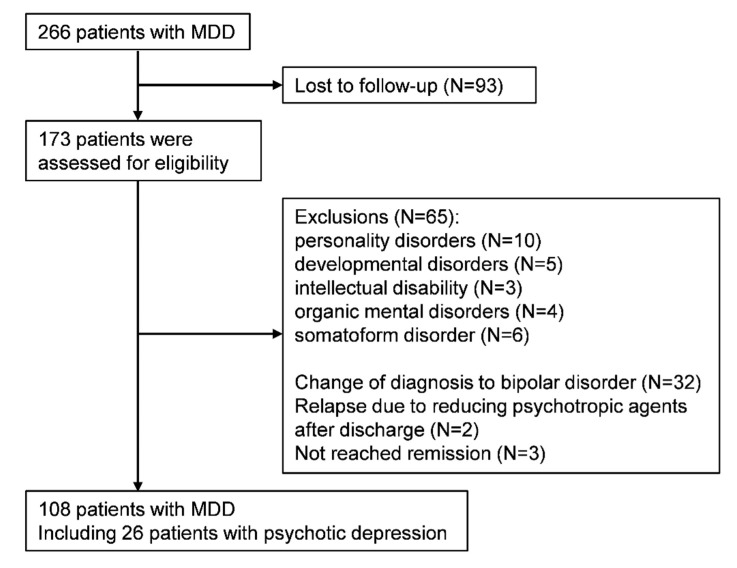
Participant flow chart.

There were 266 patients hospitalized with MDD in the study period. The length of follow-up after discharge was 12 months. Ninety-three patients were not followed up. The most common reason for a lack of follow-up data was the transfer of the patient to another clinic. The remaining 173 patients were assessed for eligibility (Figure 1). Twenty-eight patients had a comorbid diagnosis of personality disorder, developmental disorder, intellectual disability, organic mental disorder or somatic symptom disorder, and were excluded. Thirty-two patients were diagnosed with bipolar disorder after discharge from hospital. Three patients had not reached remission at discharge, and were excluded. Two patients relapsed due to reducing medication with antidepressants, and were excluded. Thus, 108 patients were included in the analysis. These 108 patients were all followed-up for 12 months. Among them, 26 patients had been diagnosed with severe MDD with psychotic features. 

Baseline characteristics were similar in patients who received benzodiazepines (BZD, *n* = 83) and patients who did not (NO-BZD, *n* = 25). This was also the same in patients with psychotic depression who received benzodiazepines (BZD-P, *n* = 14) and patients with psychotic depression who did not (NO-BZD-P, *n* = 12). Participant characteristics are provided in Table 1. *p* values are provided in Supplemental Table S1. There was no significant difference between groups of patients with MDD with psychotic features with respect to age, severity assessed by Hamilton Rating Scale for Depression (HAMD-17), time from onset to hospitalization, use of antidepressants and antipsychotics, modified electroconvulsive therapy (mECT), and the length of hospitalization.

There were no adverse effects, such as delirium or falls, reported during the administration of the benzodiazepines. Details of the benzodiazepines are provided in Supplemental Table S2. Details of the antidepressants and antipsychotics are provided in Supplemental Table S3. The acute dose at discharge was maintained throughout the follow-up period. In patients who received modified electroconvulsive therapy (mECT), benzodiazepine treatment was started after mECT.

### 3.2. Time to Depression Relapse/Recurrence

**Figure 2:**
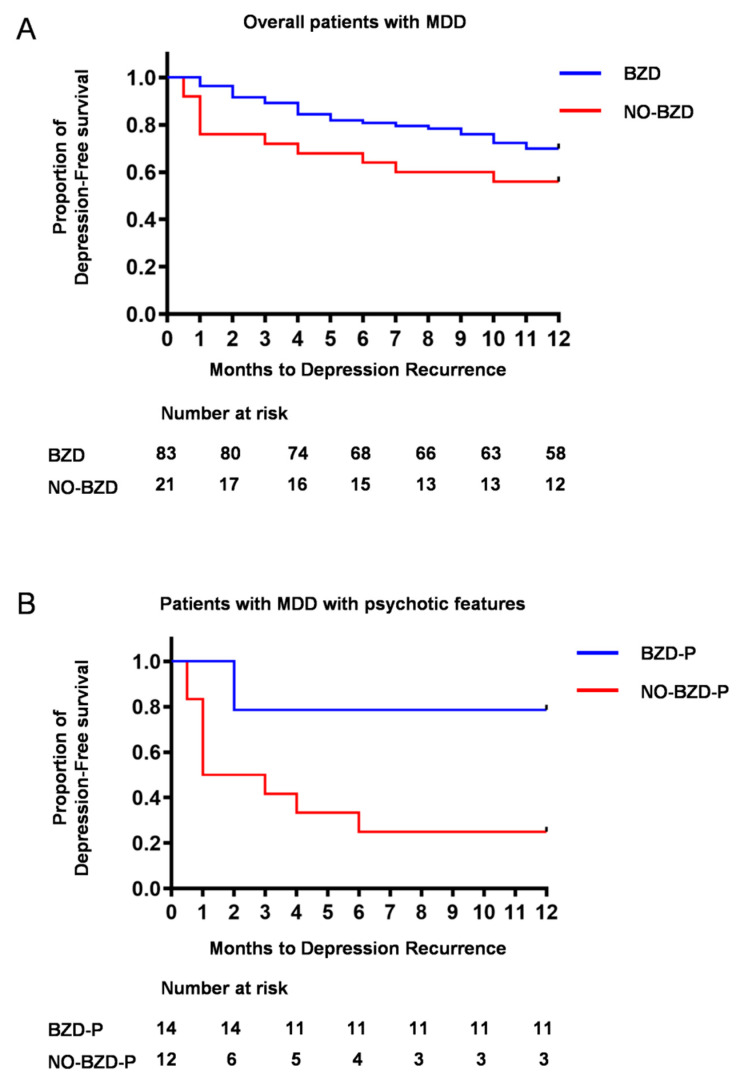
Time to depression relapse/recurrence. The cumulative incidence of relapse/recurrence was investigated by Kaplan–Meier survival analysis. (**A**) All patients with major depressive disorder. (**B**) Patients with major depressive disorder with psychotic features. BZD, patients who received benzodiazepines; NO-BZD, patients who did not receive benzodiazepines; BZD-P, patients with psychotic depression who received benzodiazepines; NO-BZD-P, patients with psychotic depression who did not receive benzodiazepines.

Kaplan–Meier survival analysis showed no significant difference in the rate of relapse/recurrence between BZD and NO-BZD (hazard ratio (95% confidence interval): 1.756 (0.776–3.973), *p* = 0.1089; Figure 2A), but did show a higher rate of relapse/recurrence in NO-BZD-P than in BZD-P (hazard ratio (95% confidence interval): 5.141 (1.591–16.61), *p* = 0.0040; Figure 2B). At the end of the 12 month follow-up, 75.0% of the NO-BZD-P group had relapsed, compared with 21.2% of the BZD-P group.

Further analyses were conducted using the Cox regression model for medications in patients with psychotic depression (Table 2). The use of benzodiazepines remained significantly associated with reduced depression recurrence (adjusted hazard ratio (95% confidence interval): 8.340 (1.621–41.92), *p* = 0.011). There were no significant covariates in antidepressants and antipsychotics (Table 2).

### 3.3. Dose-Dependent Effect of Benzodiazepines on Time to Depression Relapse/Recurrence

**Figure 3:**
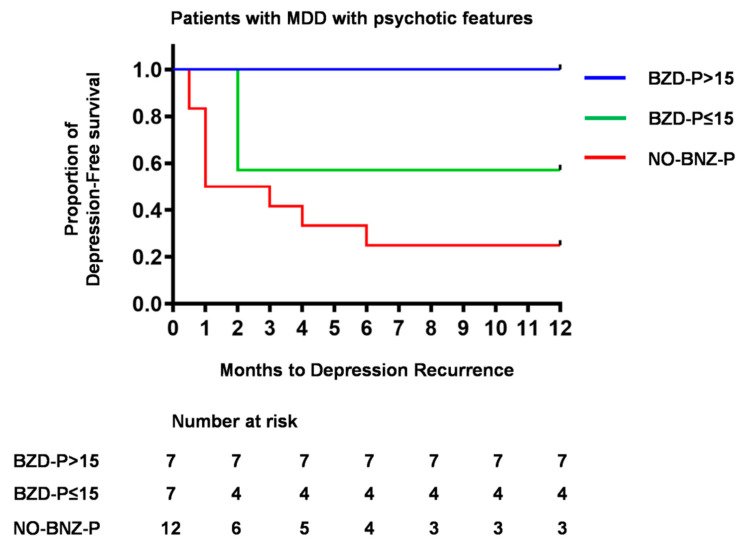
Dose-dependent effect of benzodiazepines on time to depression relapse/recurrence. Patients with major depressive disorder with psychotic features were divided into three groups: no benzodiazepine use (NO-BZD-P), benzodiazepine use with BZD score > 15 (BZD-P > 15) and benzodiazepine use with BZD score ≤ 15 (BZD-P ≤ 15).

To compare relapse/recurrence between patients receiving a high and low dose of benzodiazepines, the BZD-P group was divided into two subgroups according to benzodiazepine score (BZD score) (BZD score ≤15, BZD score >15). Kaplan–Meier survival analysis showed a significant difference in the rate of relapse/recurrence between NO-BZD-P, BZD-P with BZD score ≤15 and BZD-P with BZD score >15 (Figure 3, *p* = 0.0073). 

### 3.4. Relapse/Recurrence in Psychotic Depression

**Figure 4:**
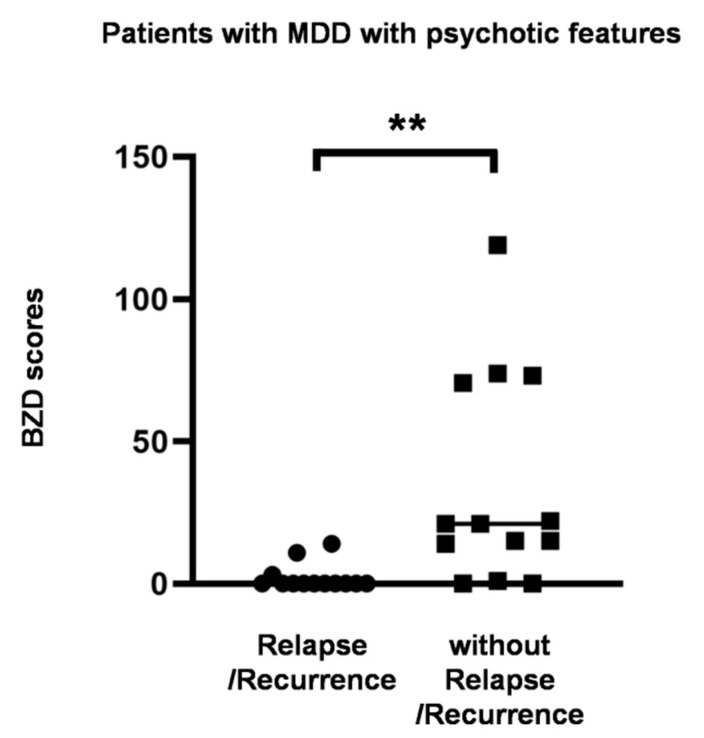
BZD score in patients with psychotic depression with and without relapse/recurrence. BZD scores were compared between those with and without relapse/recurrence using the Mann–Whitney U test. ** *p* < 0.001.

Consistent with the results of the Kaplan–Meier analysis, the BZD score was significantly higher in patients without relapse/recurrence than in patients with relapse/recurrence (Figure 4, *p* = 0.0002). Other characteristics were not significantly different (Table 3).

### 3.5. A Case Report

Some cases obviously required benzodiazepines for the maintenance of remission. For example, a 78 year old man experienced repeated recurrences within a year when only antidepressants were used, but maintained remission for years when 1 mg lorazepam was added to antidepressants. When benzodiazepines were discontinued, he experienced a recurrence of psychotic depression within a week. This recurrence was not a withdrawal symptom, because restarting lorazepam did not relieve the depressive and psychotic symptoms.

## 4. Discussion

The results of this study indicate that there is an association between the use of benzodiazepines and the relapse/recurrence rate in patients with severe MDD with psychotic features. However, benzodiazepines did not have a significant effect on relapse/recurrence when all participants were analyzed as a single group. This suggests that benzodiazepines were not effective as an adjunctive therapy for the majority of patients with moderate MDD. This is consistent with the fact that most guidelines for the clinical therapy of depression do not recommend the use of benzodiazepines [9,15].

There are several potential neurobiological mechanisms underlying the importance of benzodiazepines in MDD: (1) a reduced concentration of γ-aminobutyric acid (GABA) and altered expression of GABA receptors have repeatedly been reported in patients with MDD [16,17,18], (2) GABAergic interneuron deficits have been observed in patients with MDD [16,19], and (3) dysfunction of GABAergic interneurons is sufficient to induce depressive phenotypes in mice [20]. Furthermore, a low concentration of GABA in the cerebrospinal fluid was associated with the increased severity of psychosis [21]. Patients with severe MDD with psychotic features usually present significant anxiety symptoms; therefore, it is reasonable for GABAergic dysregulation to be found in patients with psychotic depression. It has also been reported that benzodiazepines are more often prescribed in patients with psychotic depression than in patients with MDD without psychosis [22]. Clonazepam, a long-acting benzodiazepine, significantly elongated the remission time in patients with recurrent depression [23]. Collectively, these findings support our proposal that there are some patients for whom benzodiazepines are useful.

It is also of note that we did not find reports of any adverse effects of benzodiazepines during the follow-up period. This is consistent with the opinion that the adverse effects of benzodiazepines should be evaluated when they are properly used [24].

There are several limitations of this study. First, the study was not prospective, and participants were not randomized. Second, the use of antidepressants may be different between groups. However, the differences were not significant, and Cox regression analysis revealed the use of benzodiazepines remained significantly associated with reduced depression recurrence (Table 2 and Supplemental Table S1). Furthermore, the majority of patients received the maximum dose of antidepressants approved in Japan; thus, there is little possibility that relapse/recurrence was due to the insufficient administration of antidepressants (Supplemental Table S3). Third, although there was no significant difference in the ratio between repetitive depression and first episode depression, distinguishing these populations may be better to simplify participants, which was difficult due to the small sample size. A strength of this study is the accuracy of the identified duration from remission to recurrence, due to the fact that the time of discharge was the start point of the analysis. 

## 5. Conclusions

There was no significant effect on the relapse/recurrence rate in the majority of patients with moderate MDD. Benzodiazepines should be used extremely carefully and minimally to prevent adverse effects. Nevertheless, it is important to recognize them as a therapeutic approach that may be effective against refractory recurrent cases of severe psychotic MDD.

In conclusion, benzodiazepines may contribute to a reduction in the relapse and recurrence rate over the course of 1 year in patients with severe MDD with psychotic features. A multicenter randomized controlled trial is necessary to confirm the effect of benzodiazepines on psychotic depression.

## Figures and Tables

**Table 1:** Baseline characteristics of patients.

	Overall Patients with MDD (*N* = 108)		Patients with MDD with Psychotic Features (*N* = 26)	
Characteristic	BZD (*N* = 83: Patients Who Received BZDs)	NO-BZD (*N* = 25: Patients Who Did Not Received BZDs)	BZD-P (*n* = 14: Patients Who Received BZDs)	NO-BZD-P (*n* = 12: Patients Who Did Not Received BZDs)
	*N* (%)	*N* (%)	*N* (%)	*N* (%)
Female sex	57 (68.7%)	17 (68.0%)	11 (78.5%)	10 (83.3%)
antidepressants				
SSRIs	34 (41.0%)	11 (44.0%)	9 (64.3%)	5 (41.7%)
SNRIs	15 (18.1%)	7 (28.0%)	3 (21.4%)	5 (41.7%)
mirtazapine	36 (43.4%)	7 (28.0%)	7 (50.0%)	3 (25.0%)
antipsychotics	36 (43.4%)	11 (44.0%)	7 (50.0%)	6 (50.0%)
olanzapine	8 (9.6%)	3 (12.0%)	2 (14.3%)	2 (16.7%)
quetiapine	19 (22.9%)	7 (28.0%)	4 (28.6%)	4 (33.3%)
mECT	13 (15.7%)	11 (44.0%)	10 (71.4%)	9 (75.0%)
Diagnosis				
296.2x	29 (34.9%)	9 (36.0%)		
296.21/296.22	19 (22.9%)	2 (8.0%)		
296.23	5 (6.0%)	1 (4.0%)		
296.24	4 (4.8%)	6 (24.0%)	4 (28.6%)	6 (50.0%)
296.3x	54 (65.1%)	16 (64.0%)		
296.31/296.32	32 (38.6%)	5 (20.0%)		
296.33	8 (9.6%)	5 (20.0%)		
296.34	10 (12.0%)	6 (24.0%)	10 (71.4%)	6 (50.0%)
	Mean (SD)	Mean (SD)	Mean (SD)	Mean (SD)
age	63.9 (13.2)	62.5 (16.4)	71.2 (9.79)	72.7 (10.0)
	Median (IQR)	Median (IQR)	Median (IQR)	Median (IQR)
Period from onset	6.5 (2.0~13.8)	4.0 (0.5~12.5)	7.0 (3.0~14.0)	3.0 (0.25~12.8)
	Mean (SD)	Mean (SD)	Mean (SD)	Mean (SD)
HAM-D	23.5 (5.1)	26.9 (7.28)	33.1 (2.8)	32.6 (2.7)
	Median (IQR)	Median (IQR)	Median (IQR)	Median (IQR)
Length of hospitalization (months)	2.0 (2–3)	2.0 (1.5–3)	3.0 (2–3)	2.75 (2–3.5)

MDD, major depressive disorder; BZDs, benzodiazepines; SSRI, selective serotonin reuptake inhibitor; SNRI, serotonin and norepinephrine reuptake inhibitor; mECT, modified electroconvulsive therapy; SD, standard deviation; IQR, interquartile range; BZD, patients who received benzodiazepines; NO-BZD, patients who did not receive benzodiazepines; BZD-P, patients with psychotic depression who received benzodiazepines; NO-BZD-P, patients with psychotic depression who did not receive benzodiazepines.

**Table 2:** Cox proportional hazards regression model of the effects of medications on relapse/recurrence of depression.

Parameter	Adjusted HR	95% CI	*p*-Value
benzodiazepines	8.340	1.621–41.92	0.011
SSRI	0.313	0.028–3.424	0.341
SNRI	0.488	0.043–5.504	0.561
mirtazapine	5.784	0.680–49.26	0.108
olanzapine	1.125	0.244–5.189	0.880
quetiapine	3.552	0.614–20.57	0.157

Hazard ratios were adjusted for all other variables in the table. HR, hazard ratio; CI, confidence interval; SSRI, selective serotonin reuptake inhibitor; SNRI, serotonin and norepinephrine reuptake inhibitor.

**Table 3:** Baseline characteristics of patients.

	Patients with MDD with Psychotic Features (*N* = 26)		
Characteristic	Relapse/Recurrence (*N* = 13)	Without Relapse/Recurrence (*N* = 13)	
	*N* (%)	*N* (%)	*p*-Value
Female sex	11 (84.6%)	10 (76.9%)	>0.999
antidepressants			
SSRIs	7 (53.9%)	7 (53.9%)	>0.999
SNRIs	5 (38.5%)	3 (23.1%)	0.673
mirtazapine	2 (15.4%)	8 (61.5%)	0.0414
antipsychotics	8 (61.5%)	5 (38.5%)	0.434
olanzapine	4 (30.8%)	0 (0.0%)	0.0957
quetiapine	3 (23.1%)	5 (38.5%)	0.673
mECT	9 (69.2%)	11 (75.6%)	0.678
diagnosis			
296.24	5 (38.5%)	8 (61.4%)	0.434
296.34	8 (61.4%)	5 (38.5%)	0.434
	Mean (SD)	Mean (SD)	
age	75.2 (8.54)	68.7 (10.1)	0.0777
	Median (IQR)	Median (IQR)	
Period from onset	2.0 (0.5~11.5)	5.0 (1.5~14.0)	0.340
	Mean (SD)	Mean (SD)	
HAM-D	32.8 (2.66)	32.8 (2.81)	0.946
	Median (IQR)	Median (IQR)	
Duration of hospitalization (months)	2.5 (2–3)	3.0 (2–4)	0.476

MDD, major depressive disorder; BZDs, benzodiazepines; SSRI, selective serotonin reuptake inhibitor; SNRI, serotonin and norepinephrine reuptake inhibitor; mECT, modified electroconvulsive therapy; SD, standard deviation; IQR, interquartile range. Patient characteristics were compared between those with and without relapse/recurrence using a Mann–Whitney U test and Fisher’s exact test. *p* < 0.01 was regarded as significant.

## Supplementary Materials

The following are available online at https://www.mdpi.com/2077-0383/9/6/1938/s1; Table S1: *p*-Values for table 1; Table S2. Details of benzodiazepines analyzed in this study and their half life and drug strength. Drug strength was obtained from the table of dose equivalence of benzodiazepines in a previous report [14]; Table S3. Details of antidepressants and antipsychotics.

## References

[1] Keller M.B., Trivedi M.H., Thase M.E., Shelton R.C., Kornstein S.G., Nemeroff C.B., Friedman E.S., Gelenberg A.J., Kocsis J.H., Dunner D.L. (2007). The prevention of recurrent episodes of depression with venlafaxine for two years (prevent) study: Outcomes from the 2-year and combined maintenance phases. J. Clin. Psychiatry.

[2] Reimherr F.W., Amsterdam J.D., Quitkin F.M., Rosenbaum J.F., Fava M., Zajecka J., Beasley C.M., Michelson D., Roback P., Sundell K. (1998). Optimal length of continuation therapy in depression: A prospective assessment during long-term fluoxetine treatment. Am. J. Psychiatry.

[3] Sim K., Lau W.K., Sim J., Sum M.Y., Baldessarini R.J. (2015). Prevention of relapse and recurrence in adults with major depressive disorder: Systematic review and meta-analyses of controlled trials. Int. J. Neuropsychopharmacol..

[4] Lader M. (2011). Benzodiazepines revisited--will we ever learn?. Addiction.

[5] Cumming R.G., Le Couteur D.G. (2003). Benzodiazepines and risk of hip fractures in older people: A review of the evidence. CNS Drugs.

[6] Billioti de Gage S., Begaud B., Bazin F., Verdoux H., Dartigues J.F., Peres K., Kurth T., Pariente A. (2012). Benzodiazepine use and risk of dementia: Prospective population based study. BMJ (Clin. Res. Ed.).

[7] Billioti de Gage S., Moride Y., Ducruet T., Kurth T., Verdoux H., Tournier M., Pariente A., Begaud B. (2014). Benzodiazepine use and risk of alzheimer’s disease: Case-control study. BMJ (Clin. Res. Ed.).

[8] Weich S., Pearce H.L., Croft P., Singh S., Crome I., Bashford J., Frisher M. (2014). Effect of anxiolytic and hypnotic drug prescriptions on mortality hazards: Retrospective cohort study. BMJ (Clin. Res. Ed.).

[9] National Collaborating Centre for Mental Health (2010). National Collaborating Centre for Mental Health. National institute for health and clinical excellence: Guidance. Depression: The Treatment and Management of Depression in Adults (Updated Edition).

[10] Ogawa Y., Takeshima N., Hayasaka Y., Tajika A., Watanabe N., Streiner D., Furukawa T.A. (2019). Antidepressants plus benzodiazepines for adults with major depression. Cochrane Database Syst. Rev..

[11] Benasi G., Guidi J., Offidani E., Balon R., Rickels K., Fava G.A. (2018). Benzodiazepines as a monotherapy in depressive disorders: A systematic review. Psychother. Psychosom..

[12] Bushnell G.A., Sturmer T., Gaynes B.N., Pate V., Miller M. (2017). Simultaneous antidepressant and benzodiazepine new use and subsequent long-term benzodiazepine use in adults with depression, united states, 2001–2014. JAMA Psychiatry.

[13] Manthey L., Giltay E.J., van Veen T., Neven A.K., Zitman F.G., Penninx B.W. (2011). Determinants of initiated and continued benzodiazepine use in the netherlands study of depression and anxiety. J. Clin. Psychopharmacol..

[14] Inada T., Inagaki A. (2015). Psychotropic dose equivalence in japan. Psychiatry Clin. Neurosci..

[15] Kennedy S.H., Lam R.W., McIntyre R.S., Tourjman S.V., Bhat V., Blier P., Hasnain M., Jollant F., Levitt A.J., MacQueen G.M. (2016). Canadian network for mood and anxiety treatments (canmat) 2016 clinical guidelines for the management of adults with major depressive disorder: Section 3. Pharmacological treatments. Can. J. Psychiatry Rev. Can. Psychiatr..

[16] Duman R.S., Sanacora G., Krystal J.H. (2019). Altered connectivity in depression: Gaba and glutamate neurotransmitter deficits and reversal by novel treatments. Neuron.

[17] Hasler G., van der Veen J.W., Tumonis T., Meyers N., Shen J., Drevets W.C. (2007). Reduced prefrontal glutamate/glutamine and gamma-aminobutyric acid levels in major depression determined using proton magnetic resonance spectroscopy. Arch. Gen. Psychiatry.

[18] Price R.B., Shungu D.C., Mao X., Nestadt P., Kelly C., Collins K.A., Murrough J.W., Charney D.S., Mathew S.J. (2009). Amino acid neurotransmitters assessed by proton magnetic resonance spectroscopy: Relationship to treatment resistance in major depressive disorder. Biol. Psychiatry.

[19] Fee C., Banasr M., Sibille E. (2017). Somatostatin-positive gamma-aminobutyric acid interneuron deficits in depression: Cortical microcircuit and therapeutic perspectives. Biol. Psychiatry.

[20] Fuchs T., Jefferson S.J., Hooper A., Yee P.H., Maguire J., Luscher B. (2017). Disinhibition of somatostatin-positive gabaergic interneurons results in an anxiolytic and antidepressant-like brain state. Mol. Psychiatry.

[21] Orhan F., Fatouros-Bergman H., Goiny M., Malmqvist A., Piehl F., Cervenka S., Collste K., Victorsson P., Sellgren C.M., Flyckt L. (2018). Csf gaba is reduced in first-episode psychosis and associates to symptom severity. Mol. Psychiatry.

[22] Dold M., Bartova L., Kautzky A., Porcelli S., Montgomery S., Zohar J., Mendlewicz J., Souery D., Serretti A., Kasper S. (2019). Psychotic features in patients with major depressive disorder: A report from the european group for the study of resistant depression. J. Clin. Psychiatry.

[23] Fava G.A., Ruini C., Rafanelli C., Finos L., Conti S., Grandi S. (2004). Six-year outcome of cognitive behavior therapy for prevention of recurrent depression. Am. J. Psychiatry.

[24] Balon R., Starcevic V., Silberman E., Cosci F., Dubovsky S., Fava G.A., Nardi A.E., Rickels K., Salzman C., Shader R.I. (2020). The rise and fall and rise of benzodiazepines: A return of the stigmatized and repressed. Rev. Bras. Psiquiatr..

