# Supplementary material for: Benzodiazepines Reduce Relapse and Recurrence Rates in Patients with Psychotic Depression
Source: J Clin Med. 2020 Jun 21;9(6):1938. doi: 10.3390/jcm9061938 (PMC7356546; doi:10.3390/jcm9061938)
Supplement: Supplementary file 1 [file jcm-09-01938-s001.pdf]

Supplemental Table S1. *p*-Values for table 1.

Characteristic	Overall patients with MDD (N=108)		Patients with MDD with psychotic features (N=26)	
	BZD (N=83: patients who received BZDs)	NO-BZD (N=25: patients who did not received BZDs)	BZD-P (n=14: patients who received BZDs)	NO-BZD-P (n=12: patients who did not received BZDs)
	P value		*P* value	
Female sex	>0.999		>0.999	
antidepressants				
SSRIs	0.820		0.431	
SNRIs	0.274		0.401	
mirtazapine	0.244		0.248	
antipsychotics	>0.999		>0.999	
olanzapine	0.714		>0.999	
quetiapine	0.601		>0.999	
mECT	* 0.0053		>0.999	
Diagnosis				
296.2x	>0.999			
296.21/296.22	0.149			
296.23	>0.999			
296.24	* 0.0096		0.422	
296.3x	>0.999			
296.31/296.32	0.983			
296.33	0.173			
296.34	0.196		0.422	
age	0.0502		0.675	
Period from onset	0.258		0.313	
HAM-D	0.0274		0.712	
Length of hospitalization	0.594		0.989	

MDD, major depressive disorder; BZDs, benzodiazepines; SSRI, selective serotonin reuptake inhibitor; SNRI, serotonin and norepinephrine reuptake inhibitor; mECT, modified electroconvulsive therapy; SD, standard deviation; IQR, interquartile range. Patient characteristics were compared between those with and without benzodiazepines using Mann-Whitney U test and Fisher's exact test. * $P < 0.01$.

Supplemental Table S2. Details of benzodiazepines analyzed in this study and their half life and drug strength. Drug strength was obtained from the table of dose equivalence of benzodiazepines in a previous report [14].

	Half life (h)	Drug strength
alprazolam	14	0.8
brotizolam	7	0.25
zolpidem	1.78	10
nitrazepam	25.1	5
clonazepam	27	0.25
quazepam	36.6	15
lorazepam	12	1.2
lormetazepam	10	1
clotiazepam	6.3	10

flunitrazepam	7	1
loflazepate	122	1.67
zopiclone	3.9	7.5
etizolam	6	1.5
estazolam	24	2
diazepam	27	5
triazolam	2.9	0.25
rilmazafone	10.5	2
bromazepam	8	2.5
eszopiclone	5	2.5

Supplemental Table S3. Details of antidepressants and antipsychotics.

Characteristic	Patients with MDD with psychotic features (N=26) BZD-P (n=14: patients who received BZDs)	NO-BZD-P (n=12: patients who did not received BZDs)
	N (%)	N (%)
antidepressants		
SSRIs	9 (64.3%)	5 (41.7%)
sertraline(100mg)	3	2
escitalopram (20mg)	2	3
paroxetine (50mg)	2	0
SNRIs	3 (21.4%)	5 (41.7%)
duloxetine (60mg)	2	4
venlafaxine (225mg)	1	0
mirtazapine	7 (50.0%)	3 (25.0%)
mirtazapine (45mg)	3	3
mirtazapine (30mg)	4	0
antipsychotics	7 (50.0%)	6 (50.0%)
olanzapine	2 (14.3%)	2 (16.7%)
olanzapine (2.5mg)	1	0
olanzapine (5mg)	1	2
quetiapine	4 (28.6%)	4 (33.3%)
quetiapine (25 mg)	1	0
quetiapine (75-100 mg)	2	4
quetiapine (200 mg)	1	0
